# Toxicokinetics and Tissue Distribution of the Hepatotoxic Triterpenoid Saponin Pterocephin A in Rats Using the Ultra-Performance Liquid Chromatography–Tandem Mass Spectrometry (UPLC-MS/MS) Method

**DOI:** 10.3390/molecules29215044

**Published:** 2024-10-25

**Authors:** Yiran Xiong, Zhaoyue Dong, Hongxu Zhou, Jingxin Mao, Lingjiang Zeng, Yunbin Jiang, Fancheng Meng, Zhihua Liao, Min Chen

**Affiliations:** 1 Chongqing Key Laboratory of New Drug Screening from Traditional Chinese Medicine, Integrative Science, Center of Germplasm Creation in Western China (Chongqing) Science City & Southwest University, SWU-TAAHC Medicinal Plant Joint R&D Centre, College of Pharmaceutical Sciences, Southwest University, Chongqing 400715, China; raninside@163.com (Y.X.); dzy085050@email.swu.edu.cn (Z.D.); zhouhongxu@cqifdc.org.cn (H.Z.); yunbinjiang@swu.edu.cn (Y.J.); mengfc@swu.edu.cn (F.M.); 2 Chongqing Key Laboratory of High Active Traditional Chinese Drug Delivery System, Chongqing Medical and Pharmaceutical College, Chongqing 401331, China; mmm518@163.com; 3 Chongqing Institute for Food and Drug Control, Chongqing 401120, China; 4 Integrative Science Center of Germplasm Creation in Western China (Chongqing) Science City & Southwest University, School of Life Sciences, Southwest University, Chongqing 400715, China; zengling@swu.edu.cn (L.Z.); zhliao@swu.edu.cn (Z.L.)

**Keywords:** pterocephin A, toxicokinetics, tissue distribution, UPLC-MS/MS, *Pterocephalus hookeri*

## Abstract

Pterocephin A is a natural triterpenoid saponin isolated from *Pterocephalus hookeri*, a traditional Tibetan medicine with slight toxicity, which can induce liver injury in rats. This study aimed to establish a sensitive and reliable UPLC-MS/MS method for exploring the toxicokinetics and tissue distribution of pterocephin A following single intravenous and intragastric administration. Pterocephin A and prosapogenin 1C (internal standard, IS) were extracted using a simple protein precipitation technique with methanol as the precipitant for plasma samples and methanol/acetonitrile = 1:1 (*v*/*v*) for tissue samples. UPLC separation was achieved by gradient elution with 0.3 mL/min and a mobile phase consisting of 5 mM ammonium formate (A) and acetonitrile (B) (0–2 min 30% B; 2–4 min: 30–80% B; 4–5 min: 80–98% B; 5–6.5 min: 98% B; 6.5–7 min: 98–30% B; and 7–8 min: 30% B, *v*/*v*) with a column temperature of 35 °C. MS spectrometry adopted negative ion scanning mode, primary MS spectrometry adopted full scan monitoring mode, and secondary MS spectrometry adopted targeted MS2 scan monitoring mode. The assay exhibited a linear dynamic range of 0.02–15 μg/mL for pterocephin A in biological samples, with the low limit of quantification set at 0.02 μg/mL. Non-compartmental toxicokinetic parameters indicated that pterocephin A was well absorbed into the systemic circulation and had a long residual time after intravenous (10 mg/kg) and intragastric (60 mg/kg) administration, as it could still be detected after 72 h. Tissue distribution analysis revealed detectable levels of pterocephin A in various tissues, and a high concentration was maintained in the liver after intravenous (10 mg/kg) administration, with the highest concentration being 610.95 ± 25.73 ng/mL and a specific distribution pattern of liver > lung > kidney > intestine > spleen > testes > heart > stomach. The toxicokinetic process and tissue distribution characteristics of pterocephin A were expounded in this study, which can provide relevant data support for further research and clinical application of pterocephin A with its slight toxicity.

## 1. Introduction

*Pterocephalus hookeri* (C.B. Clarke) Höeck, belonging to the family Dipsacaceae, is a traditional Tibetan medicine documented in the *Chinese Pharmacopoeia* (2020 version) [1] as a treatment for fever and rheumatoid arthritis [2,3]. Previous studies have identified the types of chemical constituents of *P. hookeri* as iridoids [4,5,6,7], triterpenoid saponins [8], and lignans [9,10]. Among them, iridoid glycosides may be the active substances with anti-rheumatoid arthritic properties [11,12,13,14]. Rheumatoid arthritis, as a chronic inflammatory disease, requires long-term medication and has higher requirements for drug safety. While classical medicine and the current pharmacopeia [15,16] have reported that it has “slight toxicity”, the research is scarce. Currently, the toxicity reports of *P. hookeri* are limited to preliminary acute toxicity studies. One study indicated that, after receiving 3000 times the clinical dose of the water extract of *P. hookeri*, mice ate less and lost weight significantly, indicating that *P. hookeri* has a certain degree of toxicity; however, the study was not thorough [17]. Therefore, in order to lay the foundation for the further development and rational clinical use of *P. hookeri*, it is necessary to conduct more in-depth research on its “small toxicity”.

Our previous study demonstrated that the n-butanol extract of *P. hookeri* constitutes the toxic fraction responsible for inducing liver injury in mice [18]. Subsequently, a new triterpenoid saponin, pterocephin A (PA), was isolated from *P. hookeri*, which might be the main toxic constituent that induces liver injury by the activation of necroptosis and inflammation [19]. Triterpenoid saponins, as glycosidic ligands of saponins, are widely distributed in nature and have a wide range of pharmacological activities. In recent years, pharmacokinetic studies of triterpenoid saponins have received much attention [20]. Jian, Yu et al. employed ESI-LC-MS/MS to establish a pharmacokinetic method for measuring various saponins in rats and revealed that oleanolic acid-type ginsenosides had high exposure levels and a slow clearance rate, which showed that their structures were similar to PA [21]. Yaqing, Ye et al. utilized an HPLC-QQQ-MS/MS method to reveal that compound AB4 exhibited low exposure levels and bioavailability of less than 1% in vivo [22]. Additionally, Yanhong, Li et al. revealed that saponins rapidly enter the bloodstream and exhibit high exposure levels, but their half-lives vary considerably and all compounds exhibited significant accumulation in the liver [23]. These studies demonstrated the feasibility of utilizing liquid chromatography and mass spectrometry for pharmacokinetic investigations in vivo. Furthermore, most triterpenoid saponins have the characteristics of high exposure, low clearance, and low bioavailability.

Therefore, as part of our ongoing research efforts, this study aimed to establish an efficient and accurate UPLC-MS/MS method for quantifying the content of PA in vivo, followed by method validation. Then, we explored the toxicokinetics and tissue distribution of PA in SD rats following both intravenous (iv) and intragastric (ig) administration to elucidate the mechanisms underlying its toxic effects and contribute to drug safety evaluations.

## 2. Results

### 2.1. Method Optimization

#### 2.1.1. Optimization of UPLC-MS/MS Conditions

**Figure 1:**
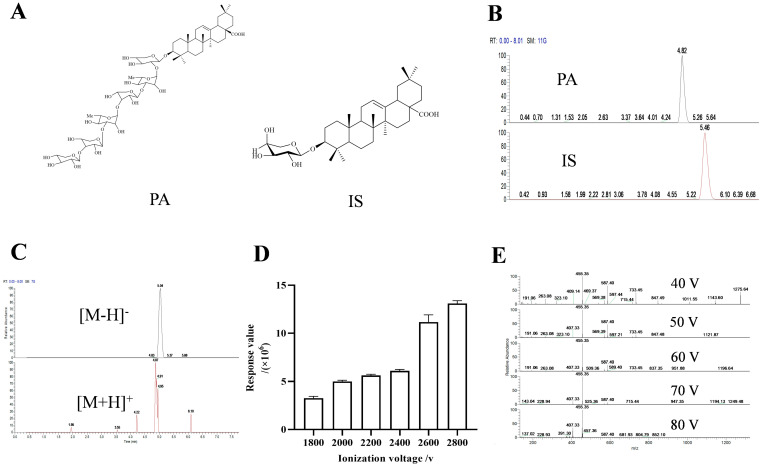
The structures of PA and IS (**A**). Chromatograms of PA and IS under acetonitrile-5 mM ammonium format (**B**). Chromatograms of PA under positive and negative ion scanning modes (**C**). Bar chart of response values of PA under different ionization voltages (**D**). Secondary mass spectra of PA under different crushing voltages (**E**).

We initially investigated the impact of different diluents and mobile phases. The findings indicated that when methanol was used as the diluent, the combination of methanol/0.1% ammonia water (*v*/*v*) and acetonitrile/5 mM ammonium formate water as the mobile phases exhibited the highest responsiveness (SD < 5 ppm), respectively, and the retention times were deemed appropriate (Table 1). Consequently, we proceeded to examine the IS under these two aforementioned mobile phases. The result showed that when acetonitrile/5 mM ammonium formate was utilized as the mobile phase, the separation effect between IS and PA was better compared to using methanol/ammonia water (Figure 1B and Figure S2).

Subsequently, we explored the response of PA under positive and negative dual scan modes. The results revealed that it had a higher response value in negative ion mode (2.04 × 10^8^ and 3.19 × 10^5^, respectively), with a more complete chromatographic peak (Figure 1C).

Then, we studied the effects of ionization voltage and breakdown voltage. Based on the protection of the instrument, we detected various voltages ranging from 1800 V to 2800 V according to the recommended ionization voltage of the negative ion mode provided by the instrument. The results indicated that it had the maximum response value at 2800V (Figure 1D). Similarly, investigation into crushing voltage indicated that 40 V delivered optimal results (Figure 1E and Figure S3).

#### 2.1.2. Optimization of Extraction Method

Using the organic solvent protein precipitation method to study the effects of methanol, acetonitrile, acetone, formic acid, and methanol/acetonitrile = 1:1 (*v*/*v*) on pre-treatment. Comprehensive consideration of the protein precipitation effect through chromatogram and recovery rate of PA and IS contained in biological samples, the results showed that pre-treatment with methanol/acetonitrile = 1:1 (*v*/*v*) had the best protein precipitation effect in blank plasma (Figure S4A). By integrating the PA extraction chromatogram, it was found that its retention effect was better (Figure S4B) than others. As mentioned above, we found that pre-treatment of tissue samples with methanol yielded the best results. (Figure S4C,D).

### 2.2. Method Validation

#### 2.2.1. Specificity

**Figure 2:**
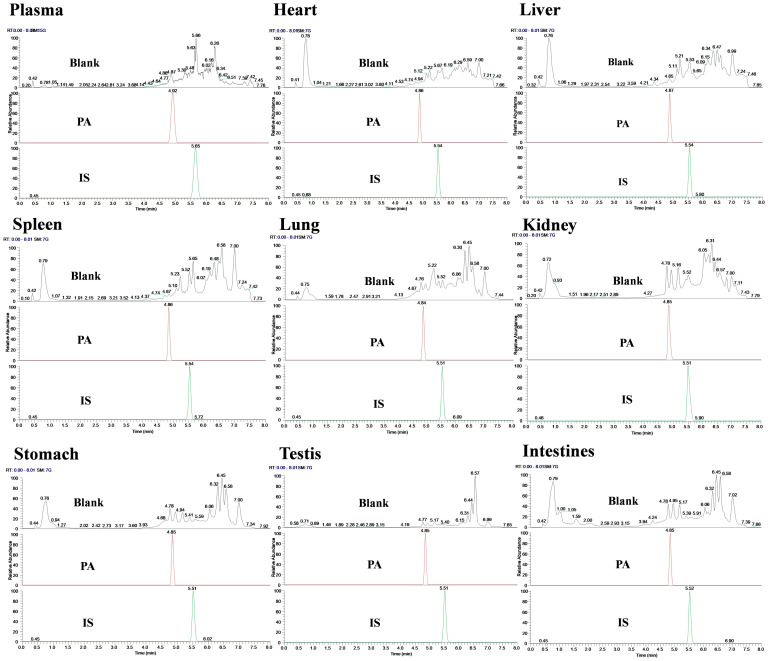
Chromatogram of specificity detection of PA and IS in various biological samples of rats.

Specificity was evaluated by comparing the chromatograms of blank and spiked samples. The results showed that the retention time of PA was approximately 4.85 min, the IS was 5.51 min, and the retention time of samples from different batches was consistent. Meanwhile, no interfering peaks were observed at PA and IS retention times or within a 10% retention window thereof under these chromatographic conditions; this indicated that there was no significant endogenous interference in the determination of biological sample content (Figure 2).

#### 2.2.2. Linearity and LLOQ

Linearity was evaluated by calculation of the regression equations through the method of least squares over the range of 0.02–15 μg/mL in plasma and tissues. The results indicated that all biological matrices had good linear regression within the detected concentration range (R^2^ > 0.9954) and the signal-to-noise ratio met the requirements. LLOQ was determined to be 0.02 μg/mL (Table 2).

#### 2.2.3. Precision and Accuracy

The precision and accuracy of PA were determined at three levels (0.02, 0.2, and 1.0 μg/mL) with five replicates at each level in all biological matrices. The recovery rate of the target analyte ranges from 96.11% to 108.93%, and the intra-day and inter-day accuracy values (RSDs) were less than 10.0%. It indicated that the method was reliable and repeatable in the quantitative analysis of PA within an acceptable range (Table 3).

#### 2.2.4. Stability

The stability of PA under different storage conditions was investigated at three QC levels and the results were summarized in Table 4. At three concentrations, the recovery rates of PA in the plasma samples were greater than 90%, the RSDs of high and low concentrations were less than 10%, and no obvious deviation values were observed, which met the requirements.

#### 2.2.5. Extraction Recovery and Matrix Effect

The matrix effects of the analyte were assessed by comparing the peak areas of the blank matrix samples spiked after extraction with the same standard solution in pure solvent. At three concentrations, the extraction recovery ranges from 96.08% to 109.91%, and the RSDs were < 10% (Table 5), indicating that endogenous or exogenous substances in plasma and tissue samples did not significantly inhibit or enhance the detection signal of PA, and did not affect its accurate quantification. The method met the experimental requirements.

### 2.3. Plasma Toxicokinetics

#### 2.3.1. Intravenous Administration TKs

**Figure 3:**
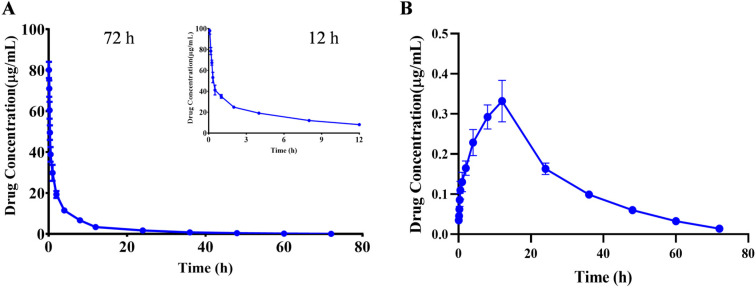
Mean plasma concentration-time profiles of PA after a single iv (**A**) and ig (**B**) administration. Results are presented as mean values ± SD (*n* = 6).

The kinetic parameters were calculated using the PKslower 2.0 pharmacokinetic analysis software based on a non-compartmental model. The results showed that after iv administered a single dose of 10 mg/kg PA, the biological half-life (t_1/2_) was 10.466 ± 1.466 h; the peak concentration (C_max_) was 80.115 ± 3.641 μg/mL; the offline area during 0–72 h of medication (AUC_all_) was 217.523 ± 18.896 μg/mL×h, and the (AUC_inf_) was 218.704 ± 19.154 μg/mL×h; the apparent distribution volume (Vz) was 0.697 ± 0.122 (mg)/(μg/mL); the clearance rate (CL) was 0.046 ± 0.004 (mg)/(μg/mL)/h; and the average residence time (MRT_0-inf_) was 9.540 ± 0.752 h (Table 6). The drug–time curve result suggested that PA had a longer clearance time after entering the body, and a small amount of compound was still detected after 72 h, which indicated a slow metabolism rate of the compound in the body, which may be due to the lack of relevant metabolic enzymes in the body and other reasons (Figure 3A and Table 6).

#### 2.3.2. Intragastric Administration TKs

After ig administration of 60 mg/kg, the toxicokinetic parameters were calculated as follows: the biological half-life (t_1/2_) was 13.797 ± 1.755 h; the peak concentration (C_max_) was 0.352 ± 0.016 μg/mL; the offline area during 0–72 h of medication (AUC_all_) was 9.253 ± 0.451 μg/mL×h, and the (AUC_inf_) was 9.539 ± 0.480 μg/mL×h; the apparent distribution volume(Vz/F) was 125.521 ± 17.130 (mg)/(μg/mL); the clearance rate (CL/F) was 6.306 ± 0.327 (mg)/(μg/mL)/h; and the average residence time (MRT_0-inf_) was 23.580 ± 1.335 h (Figure 3B and Table 6). Calculation of parameters revealed that the bioavailability was less than 1%. The reason may be attributed to the high tissue distribution and low blood levels of the compound, or due to the lower bioavailability after metabolism in the intestine or liver, resulting in poor intestinal absorption [22].

### 2.4. Tissue Distribution

**Figure 4:**
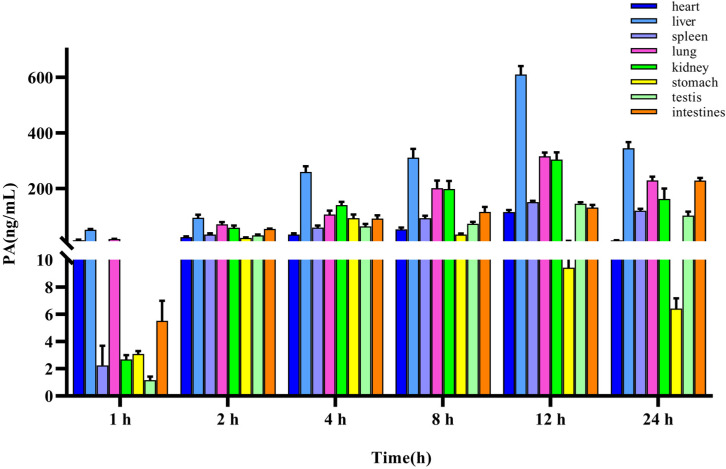
Tissue distribution of PA after a single iv administration (10 mg/kg). Results are presented as mean values ± SD (*n* = 4).

The method for quantifying PA in rat tissues was applied to a tissue distribution study in rats following the iv administration of 10 mg/kg PA. The concentration of PA in different tissues was calculated using a standard curve, and the mean-tissue-concentration–time columnar chart is shown in Figure 4. The concentrations in the heart, liver, spleen, lung, kidney, stomach, testes, and intestines were measured within 24 h. The results indicated that PA was widely distributed in various tissues of rats after entering the bloodstream, which was consistent with the findings of TK studies. The site with the highest accumulation was the liver, with the highest concentration being (610.95 ± 25.73 ng/mL), followed by the lungs (316.63 ± 11.18 ng/mL), kidney (304.54 ± 22.58 ng/mL), intestine (228.79 ± 8.23 ng/mL), spleen (151.40 ± 4.23 ng/mL), testes (145.47 ± 4.27 ng/mL), heart (115.48 ± 6.52 ng/mL), and stomach (93.72 ± 11.28 ng/mL).

Simultaneously, we analyzed the concentration changes in various organs and observed that the liver, spleen, lungs, kidneys, testes, and heart reached the highest concentration after approximately 12 h, the stomach after 4 h, and the intestine continued to rise after 24 h, indicating that tissue exposure was gradually increasing, which may be due to intestinal reabsorption and tissue redistribution.

Furthermore, a combined analysis of toxicokinetic parameters following iv administration revealed that plasma exposure rapidly decreased after PA entered the body, while tissue exposure gradually increased within 12 h. It was worth noting that after reaching a certain threshold, plasma exposure tended to stabilize, but was accompanied by a longer plasma half-life.

## 3. Materials and Methods

### 3.1. Animals

Animals specific pathogen-free (SPF)-grade male SD rats, weighing 200 ± 20 g and 6–8 weeks old, were purchased from Hunan Slake Jingda Experimental Animal Co, Ltd. (No. 430727240101622886). The rats were maintained at a constant temperature (22 ± 2 °C) in standard ventilated cages and water ad libitum, while the environment had regulated lighting (12 h of strong light, 12 h of darkness) and a relative humidity of 50% ± 10%. Animal experiments had been approved by the Ethics Review Committee for Animal Experiments at Southwest University, with the serial number (IACUC-20240604-04). During the experiment, the experimental facilities met the safety requirements, the operation was standardized, and the animals were anesthetized before they were killed to alleviate the pain and ensure their welfare.

### 3.2. Instrumentation and Analytical Conditions

UPLC-MS/MS (Thermo Scientific^TM^ Orbitrap Exploris^TM^ 240 mass spectrometer, Waltham, MA, USA) was used to analyze the content of PA in biological samples. Performed chromatographic separation by using a Hypersil GOLD C18 column (2.1 mm × 100 mm, 1.9 μm). UPLC separation was achieved by gradient elution with 0.3 mL/min and the mobile phase consisting of 5 mM ammonium formate (A) and acetonitrile (B) (0–2 min 30% B; 2–4 min: 30–80% B; 4–5 min: 80–98% B; 5–6.5 min: 98% B; 6.5–7 min: 98–30% B; and 7–8 min: 30% B, *v*/*v*), the column temperature was 35 °C, the injection volume was 3 µL, and the column temperature was set at 30 °C.

The mass spectrometric detection was conducted under full scan and targeted MS2 scan monitoring in a negative ionization mode using the following conditions: ion source type was electron spray ionization (ESI), sheath gas at 35 Arb, aux gas at 10 Arb, sweep gas at 2 Arb, ion transfer tube temperature of 350 °C, and vaporizer temperature of 350 °C. The methodology of this experiment was developed based on the above conditions.

### 3.3. Materials and Reagents

Pterocephin A (PA) and prosapogenin 1C (IS) were isolated by our research team from the n-butanol fraction of *P. hookeri*. Their structures are shown in Figure 1A, and the purity (≥98%) of all compounds was determined by NMR (Figure S1). The methanol, acetonitrile, and ammonium formate used in the experiment were purchased from ThermoFisher and all were of mass spectrometry grade. Other commercially available reagents were analytical grade.

### 3.4. Preparation of Standard Solution and Quality Control Samples

We accurately weighed 1.0 mg of PA and IS and prepared the stock and working solutions with 1.0 mg/mL with 1.0 mL of chromatographic methanol. We diluted the working solution of PA with chromatographic methanol using the gradient dilution method to concentrations of 15.0, 7.5, 3.25, 1.6, 0.8, 0.4, 0.2, 0.1, 0.05, and 0.02 μg/mL, and diluted IS to 0.1 μg/mL with a volume of 50.0 μL. Then, 50.0 μL of blank plasma was added to each concentration working solution, and the protein was precipitated by organic solvent, dried with liquid nitrogen, and then dissolved in 50.0 μL of chromatographic methanol. After centrifugation and filtration, we took the supernatant to obtain the calibration curve samples. The same procedure was used to create three concentration levels of quality control (QC) samples (0.02, 0.1, 1.0 μg/mL). While not in use, all of the working and stock solutions were kept at −20 °C.

### 3.5. Sample Preparation

The protein precipitation method was used to extract the target compound. An amount of 50.0 μL of a plasma/tissue homogenate sample was mixed with an equivalent volume of 0.1 μg/mL of IS working solution. Then, 400.0 μL of organic solvent was added to the mixed solution and vortexed for 10 min for protein precipitation; after being centrifuged at 4 °C and 4500 rpm for 15 min, the supernatant was collected and blown dry with liquid nitrogen, 50.0 μL of methanol was added for reconstitution, and then it was centrifuged again to collect the supernatant for sample detection.

### 3.6. Method Validation

#### 3.6.1. Specificity

To investigate whether the detection of PA could be affected by endogenous components in the plasma and tissues of rats. Blank plasma/tissue samples and blank plasma/tissue samples containing IS and PA were used for testing. The comparative analysis of chromatograms confirmed the exclusivity of this method in detecting PA.

#### 3.6.2. Linearity and LLOQ

Calibration curves were prepared by adding the working standard solutions into blank rat plasma and tissues to obtain calibration concentrations of 15.0, 7.5, 3.25, 1.6, 0.8, 0.4, 0.2, 0.1, 0.05, and 0.02 μg/mL, and the calibration concentrations were in the range of 0.02–15 μg/mL. Linearity was evaluated by the correlation coefficient (R^2^), and a value of at least 0.99 was considered to be acceptable. Three replications of the calibration curve were performed on the intra-day.

#### 3.6.3. Precision and Accuracy

The intra- and inter-day precision and accuracy were assessed by analyzing the LLOQ and QC samples (0.02, 0.2, 1.0 μg/mL) on one day and five consecutive days, using calibration curves established daily (*n* = 5). Precision and accuracy were calculated as the RSDs of measured concentrations. The acceptable values of RSDs for this validation were within ±15%.

#### 3.6.4. Stability

The stability of PA was investigated by analyzing five replicates of the samples at QC levels (0.02, 0.2, and 1.0 μg/mL) under different storage conditions. They were, respectively, placed for 24 h (25 °C), 7 days (−80 °C), and 3 freeze–thaw cycles (−20~25 °C) for testing, and the bias value was calculated to evaluate the stability of the method.

#### 3.6.5. Extraction Recovery and Matrix Effect

For extraction recovery and matrix effect analysis, LLOQ and QC (0.02, 0.2, 1.0 μg/mL) concentrations were applied, with 5 parallel samples for each concentration. We used the sample ratio of plasma to methanol to investigate whether there is a matrix effect in plasma samples. Similarly, the RSDs of the matrix effect should be less than 15%.

### 3.7. Toxicokinetics Study

Adaptive feeding of SPF-grade male SD rats (weighing 200 ± 20 g, *n* = 6) was performed for a week, and then they were fasted for 12 h before administration with free access to water. The administration method and dosage were iv of 10 mg/kg and ig of 60 mg/kg PA. Plasma samples were collected in heparinized Eppendorf tubes via the post-ocular vein at 0, 0.083, 0.167, 0.25, 0.5, 1, 2, 4, 8, 12, 18, 24, 48, 60, and 72 h after administration. After centrifuging at 4 °C and 4500 rpm for 10 min, the plasma samples were obtained for immediate biochemical analysis or stored at −80 °C until they were ready for the toxicokinetic studies. A non-compartmental model was used to calculate toxicokinetic parameters expressed as mean ± SD using PKslover 2.0 and Excel 2010.

### 3.8. Tissue Distribution

SPF grade male SD rats (weighing 200 ± 20 g, *n* = 24), adaptive feeding for a week, after iv administration at a dose of 10 mg/kg, 4 rats were executed at 1, 2, 4, 8, 12 and 24 h, respectively. Then, we separated the heart, liver, spleen, lung, kidney, stomach, testes, and intestinal tissues. Rinsed each with physiological saline to remove blood and mucus. Tissue samples were homogenized with 5-fold physiological saline, centrifuged at 12,000 rpm for 10 min at 4 °C, and subjected to UPLC-MS/MS testing after pre-treatment of the supernatant.

## 4. Discussion

*Pterocephalus hookeri* contains a multitude of active ingredients, and researchers have assessed the quality of its bioactive components utilizing UPLC-TQ-MS/MS [24]. Nevertheless, there have been a few studies on the toxic constituents, as well as limited investigation on the associated toxicokinetics and quantitative detection in biological samples. Our current research developed an optimized method that was characterized by short duration, high efficiency, and minimal interference through refined liquid chromatography conditions. Methodological investigations have demonstrated both feasibility and reproducibility. Concurrently, we evaluated a sample pre-treatment approach based on extraction recovery rates and protein precipitation effects. Overall, this method shows promise for application in the toxicokinetics of PA.

Applying the aforementioned method to the toxicokinetics research of PA can elucidate the mechanisms of action patterns in vivo. The results demonstrated that PA rapidly entered the bloodstream, and exhibited a prolonged residence time within the body. We also found PA has high exposure levels and low clearance in vivo. Concurrently, the investigations into tissue distribution following intravenous injection revealed significant accumulation in the liver. This observation may be attributed to the activation of protective mechanisms in the liver and kidneys, which are critical organs for metabolism and detoxification. As a result of their protective responses, these organs may accumulate higher concentrations of the compound when exposed to toxic substances. These findings suggested that PA may exhibit a degree of tissue targeting. Meanwhile, PA has better water solubility due to the presence of six sugar groups, allowing it to enter the bloodstream quickly and be distributed to various organ tissues rapidly. Overall, these findings are consistent with our prior research on PA-induced liver injury.

In recent years, research on the metabolism of pentacyclic triterpenoids in vivo has also become increasingly widespread. Research has indicated that pentacyclic triterpenoid compounds undergo deglycosylation [25,26], oxidation [27], and dehydration [28,29] reactions in vivo and exert their effects through hydrolysis to form aglycones [30,31]. Due to the high molecular weight and relatively weak intestinal absorption capacity, they are more susceptible to the influence of intestinal microbiota [32], which further leads to prolonged retention time in vivo and induces accumulation. The above research and discussion provide valuable insights for further study on the metabolism of PA in vivo and offer useful information for its clinical application.

## Figures and Tables

**Table 1:** Response value under different diluents and mobile phases (*n* = 5).

Mobile Phase	Response Values of Different Diluents		
	Methanol	Acetonitrile	Acetone
Methanol/Water	1.05 × 10^6^ ± 8.35 × 10^4^	2.78 × 10^6^ ± 2.58 × 10^5^	1.10 × 10^6^ ± 8.76 × 10^4^
Methanol/0.1% Ammonia	1.95 × 10^8^ ± 2.46 × 10^7^	5.87 × 10^7^ ± 7.04 × 10^6^	6.70 × 10^7^ ± 1.86 × 10^6^
Methanol/0.1% Formic acid water	1.77 × 10^7^ ± 2.01 × 10^6^	1.79 × 10^7^ ± 1.26 × 10^6^	1.02 × 10^7^ ± 5.59 × 10^5^
Methanol/5 mM Ammonium formate water	4.97 × 10^7^ ± 2.36 × 10^6^	2.64 × 10^7^ ± 1.80 × 10^6^	2.36 × 10^7^ ± 1.86 × 10^6^
Acetone/Water	1.63 × 10^7^ ± 1.40 × 10^6^	1.37 × 10^7^ ± 1.82 × 10^6^	9.99 × 10^6^ ± 2.57 × 10^5^
Acetone/0.1% Ammonia	5.51 × 10^7^ ± 1.95 × 10^6^	1.88 × 10^7^ ± 1.47 × 10^6^	2.25 × 10^7^ ± 1.77 × 10^6^
Acetone/0.1% Formic acid water	5.73 × 10^7^ ± 3.69 × 10^6^	5.51 × 10^7^ ± 1.94 × 10^6^	5.06 × 10^7^ ± 1.35 × 10^6^
Acetone/5 mM Ammonium formate water	6.97 × 10^7^ ± 1.37 × 10^6^	5.54 × 10^7^ ± 2.14 × 10^6^	4.75 × 10^7^ ± 1.30 × 10^6^

**Table 2:** Linear regression equations for different organizations (*n* = 5).

Samples	Linear Equations	R^2^	Linear Range (μg/mL)
Plasma	y = 0.2079x + 0.0013	0.9977	0.02–15
Heart	y = 0.2721x + 0.1382	0.9954	
Liver	y = 0.2016x + 0.1683	0.9963	
Spleen	y = 0.1688x + 0.1911	0.9960	
Lung	y = 0.1593x + 0.0185	0.9972	
Kidney	y = 0.1502x + 0.0230	0.9980	
Stomach	y = 0.1773x + 0.0149	0.9993	
Testes	y = 0.1593x + 0.0185	0.9972	
Intestines	y = 0.2234x + 0.0156	0.9994	

**Table 3:** Intra-day and inter-day precision of different organizations (*n* = 5).

Samples	Spiked Concentration (μg/mL)	Intra-Day Precision			Inter-Day Precision		
		Calculate Concentration (μg/mL)	Extraction Recovery (%)	RSD (%)	Calculate Concentration (μg/mL)	Extraction Recovery (%)	RSD (%)
Plasma	0.02	0.021 ± 0.001	106.20 ± 5.99	5.31	0.021 ± 0.002	103.15 ± 8.00	7.53
	0.2	0.194 ± 0.013	96.98 ± 6.33	6.74	0.193 ± 0.156	96.27 ± 7.78	8.39
	1.0	1.018 ± 0.089	101.80 ± 8.93	8.62	1.093 ± 0.047	107.36 ± 4.58	3.98
Heart	0.02	0.021 ± 0.002	102.84 ± 9.15	8.64	0.021 ± 0.002	105.79 ± 8.42	7.53
	0.2	0.230 ± 0.016	108.78 ± 7.39	6.24	0.214 ± 0.020	107.02 ± 10.08	8.80
	1.0	1.096 ± 0.089	106.04 ± 8.58	7.63	1.052 ± 0.084	105.22 ± 8.38	7.65
Liver	0.02	0.020 ± 0.002	101.78 ± 8.32	8.03	0.022 ± 0.001	102.67 ± 5.92	5.62
	0.2	0.215 ± 0.014	107.75 ± 7.00	6.03	0.228 ± 0.015	105.34 ± 6.84	7.57
	1.0	1.035 ± 0.082	102.05 ± 8.13	7.81	1.099 ± 0.085	103.31 ± 7.98	7.47
Spleen	0.02	0.022 ± 0.002	108.93 ± 9.46	7.97	0.021 ± 0.002	102.71 ± 8.28	7.85
	0.2	0.192 ± 0.016	96.11 ± 8.26	8.93	0.223 ± 0.019	106.52 ± 9.17	8.08
	1.0	1.150 ± 0.089	106.61 ± 8.27	7.28	0.997 ± 0.068	97.35 ± 3.21	3.39
Lung	0.02	0.019 ± 0.001	99.79 ± 4.06	4.22	0.019 ± 0.001	96.30 ± 5.11	5.45
	0.2	0.021 ± 0.015	104.36 ± 7.77	7.14	0.213 ± 0.012	100.48 ± 5.58	5.52
	1.0	1.032 ± 0.080	103.25 ± 8.03	7.54	1.089 ± 0.051	108.92 ± 5.06	4.27
Kidney	0.02	0.020 ± 0.001	99.79 ± 5.17	5.19	0.020 ± 0.001	99.90 ± 4.77	4.77
	0.2	0.211 ± 0.011	102.11 ± 5.28	4.79	0.213 ± 0.008	106.53 ± 4.21	3.71
	1.0	1.078 ± 0.060	107.75 ± 5.97	5.14	1.050 ± 0.061	104.97 ± 6.07	5.51
Stomach	0.02	0.020 ± 0.001	99.01 ± 5.76	5.88	0.022 ± 0.001	109.16 ± 4.89	4.10
	0.2	0.197 ± 0.007	98.75 ± 3.58	3.67	0.205 ± 0.008	102.37 ± 4.02	3.84
	1.0	1.070 ± 0.027	106.98 ± 2.65	2.32	1.091 ± 0.021	107.58 ± 2.05	3.79
Testes	0.02	0.021 ± 0.001	106.21 ± 6.47	5.73	0.020 ± 0.001	99.91 ± 2.41	2.41
	0.2	0.206 ± 0.008	103.03 ± 3.97	3.74	0.200 ± 0.003	99.90 ± 1.70	1.70
	1.0	0.978 ± 0.032	97.84 ± 3.20	3.34	1.086 ± 0.040	108.63 ± 3.96	3.36
Intestines	0.02	0.021 ± 0.001	102.04 ± 5.47	5.26	0.020 ± 0.001	97.76 ± 4.73	4.95
	0.2	0.202 ± 0.007	101.04 ± 3.51	3.43	0.207 ± 0.012	103.53 ± 5.92	5.53
	1.0	1.003 ± 0.055	100.34 ± 5.53	5.49	0.980 ± 0.023	97.90 ± 2.26	2.36

**Table 4:** Investigation of plasma stability (*n* = 5).

Samples	Spiked Concentration (μg/mL)	Normal Temperature		−80 °C Frozen Storage		Freeze Thawing	
		Extraction Recovery (%)	RSD (%)	Extraction Recovery (%)	RSD (%)	Extraction Recovery (%)	RSD (%)
Plasma	0.02	99.89 ± 4.27	4.28	108.61 ± 6.74	5.72	105.60 ± 8.51	7.63
	0.2	107.50 ± 4.69	4.06	105.39 ± 8.24	7.42	99.89 ± 4.24	4.29
	1.0	108.76 ± 3.15	2.67	107.69 ± 5.24	4.52	103.83 ± 5.64	5.23

**Table 5:** Matrix effect investigation (*n* = 5).

Samples	Spiked Concentration (μg/mL)	Extraction Recovery (%)	RSD (%)
Plasma	0.02	97.56 ± 3.52	3.70
	0.2	106.05 ± 4.43	3.94
	1.0	104.19 ± 7.24	6.67
Heart	0.02	103.34 ± 8.69	8.14
	0.2	99.06 ± 5.23	5.33
	1.0	98.29 ± 4.75	4.91
Liver	0.02	103.27 ± 7.54	7.07
	0.2	104.25 ± 8.69	8.00
	1.0	102.92 ± 9.10	8.59
Spleen	0.02	104.48 ± 8.06	7.38
	0.2	96.08 ± 4.76	5.16
	1.0	103.93 ± 6.36	5.89
Lung	0.02	104.36 ± 7.77	7.14
	0.2	109.29 ± 5.49	4.59
	1.0	101.99 ± 4.49	4.40
Kidney	0.02	99.65 ± 5.39	5.42
	0.2	106.64 ± 4.21	3.71
	1.0	98.27 ± 3.23	3.35
Stomach	0.02	105.93 ± 5.50	4.90
	0.2	104.47 ± 4.24	3.90
	1.0	109.91 ± 3.13	2.85
Testes	0.02	105.52 ± 6.15	5.52
	0.2	102.49 ± 4.42	4.20
	1.0	106.03 ± 2.23	2.10
Intestines	0.02	99.49 ± 5.91	5.97
	0.2	109.11 ± 6.34	5.32
	1.0	99.34 ± 3.82	3.88

**Table 6:** Pharmacokinetic parameters of PA following (IV, 10 mg/kg) and (IG, 60 mg/kg) administration in rats (mean ± SD, *n* = 6).

Parameters	Unit	IV (10 mg/kg)	IG (60 mg/kg)
t_1/2_	h	10.466 ± 1.466	13.797 ± 1.755
C_max_	μg/mL	80.115 ± 3.641	0.352 ± 0.016
AUC_all_	μg/mL×h	217.523 ± 18.896	9.253 ± 0.451
AUC_inf_	(mg)/(μg/mL)	218.704 ± 19.154	9.539 ± 0.480
Vz, Vz/F	(mg)/(μg/mL)	0.697 ± 0.122	125.521 ± 17.130
CL, CL/F	(mg)/(μg/mL)/h	0.046 ± 0.004	6.306 ± 0.327
MRT_0-inf_	h	9.540 ± 0.752	23.580 ± 1.335

Note: t_1/2_, terminal half-life; C_max_, peak plasma concentration; AUC_all_, the area under the plasma concentration vs. time curve from time zero to the time of the last observation; AUC_inf_, AUC from time zero to infinity; Vz, apparent volume of distribution; CL, clearance; MRT_0-inf_, mean residence time zero to infinity.

## Data Availability

The data presented in this study are available on request from the corresponding author. The data are not publicly available due to privacy restrictions (containing information that could compromise the privacy of research participants).

## Supplementary Materials

The following supporting information can be downloaded at: https://www.mdpi.com/article/10.3390/molecules29215044/s1, Figure S1: ^13^C-NMR of PA (A), ^1^H-NMR of PA (B), ^13^C-NMR of IS (C), ^1^H-NMR of IS (D). Figure S2: representative chromatograms of PA and IS under methanol–0.1% ammonia. Figure S3: secondary mass spectra of PA under different crushing voltages. Figure S4: chromatograms for pre-treatment examination of blank plasma (A), PA-containing plasma (B), blank tissue (C), and PA-containing tissue (D).
