# Supplementary material for: Toxicokinetics and Tissue Distribution of the Hepatotoxic Triterpenoid Saponin Pterocephin A in Rats Using the Ultra-Performance Liquid Chromatography–Tandem Mass Spectrometry (UPLC-MS/MS) Method
Source: Molecules. 2024 Oct 25;29(21):5044. doi: 10.3390/molecules29215044 (PMC11547388; doi:10.3390/molecules29215044)
Supplement: Supplementary file 1 [file molecules-29-05044-s001.zip › molecules-3240557-supplementary.pdf]

# SUPPLEMENTATY MATERIAL

## Toxicokinetics and Tissue Distribution of the Hepatotoxic Triterpenoid Saponin Pterocephin A in Rats Using the Ultra-Performance Liquid Chromatography–Tandem Mass Spectrometry (UPLC-MS/MS) Method

Yiran Xiong [1, 2], Zhaoyue Dong [1], Hongxu Zhou [1, 3], Jingxin Mao [2], Lingjiang Zeng [4], Yunbin Jiang [1], Fancheng Meng [1], Zhihua Liao [4], Min Chen [1, 2, *]

**Contents of Supplementary Information.**

**Figure S1**. $^{13}$C-NMR of PA (A), $^{1}$H-NMR of PA (B), $^{13}$C-NMR of IS (C), $^{1}$H-NMR of IS (D).

**Figure S2**. Representative chromatograms of PA and IS under methanol-0.1% ammonia.

**Figure S3**. Secondary mass spectra of PA under different crushing voltages.

**Figure S4**. Chromatograms for pre-treatment examination of blank plasma (A), PA containing plasma (B), blank tissue (C) and PA containing tissue (D).

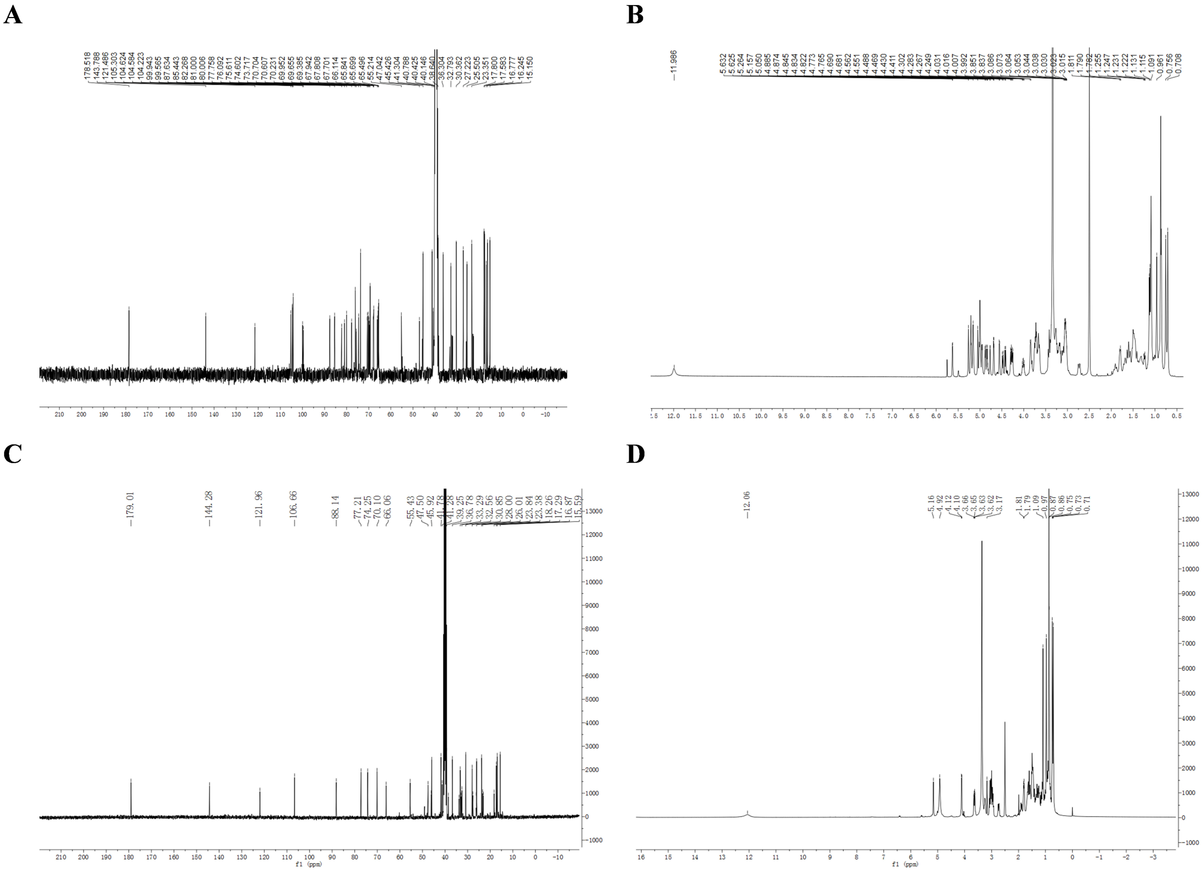


**Figure S1**. $^{13}$C-NMR of PA (A), $^{1}$H-NMR of PA (B), $^{13}$C-NMR of IS (C), $^{1}$H-NMR of IS (D).

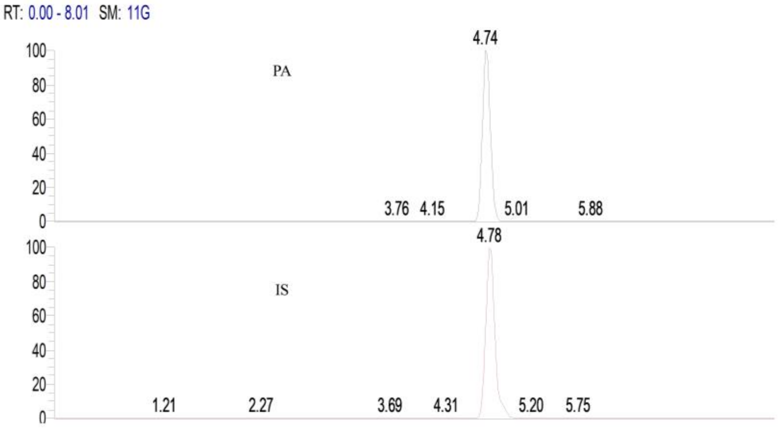


**Figure S2**. Representative chromatograms of PA and IS under methanol-0.1% ammonia.

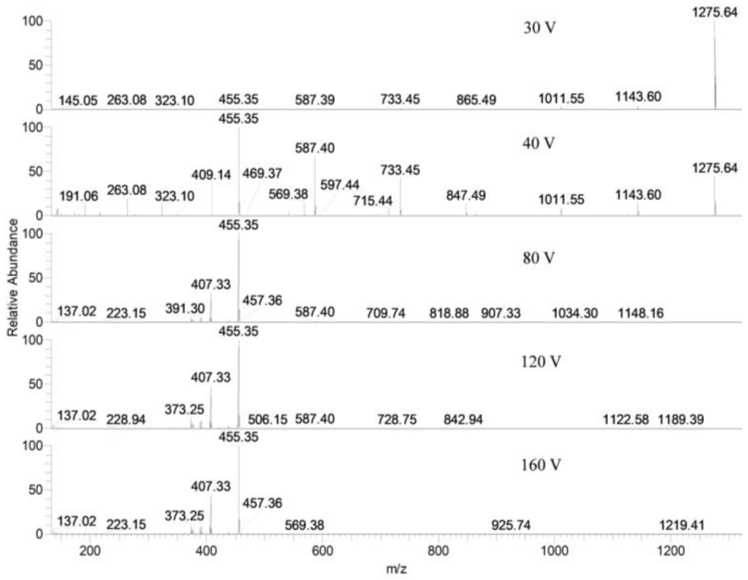


**Figure S3**. Secondary mass spectra of PA under different crushing voltages.

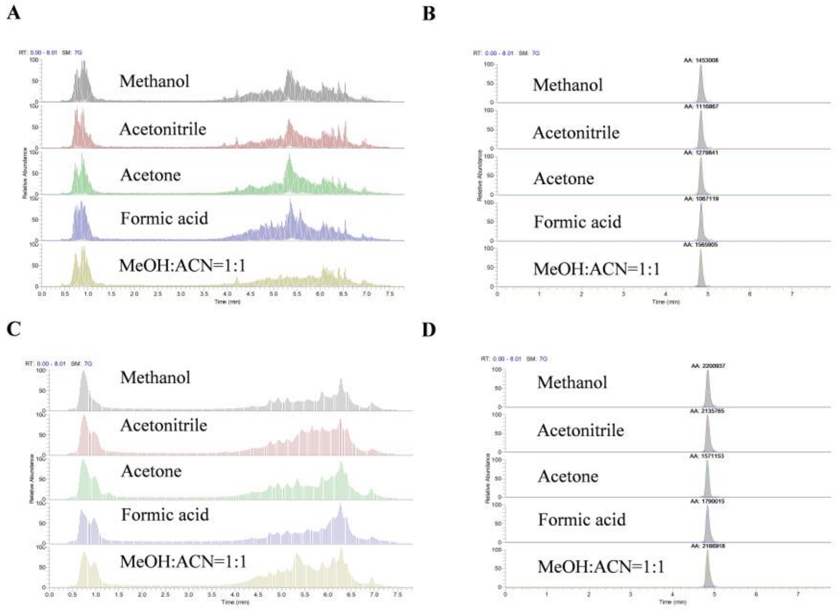


**Figure S4**. Chromatograms for pre-treatment examination of blank plasma (A), PA containing plasma (B), blank tissue (C) and PA containing tissue (D).
